# Elimination of LRVs Elicits Different Responses in *Leishmania* spp.

**DOI:** 10.1128/msphere.00335-22

**Published:** 2022-08-09

**Authors:** Andreu Saura, Alexandra Zakharova, Donnamae Klocek, Evgeny S. Gerasimov, Anzhelika Butenko, Diego H. Macedo, Elena Servienė, Diana Zagirova, Anastasia Meshcheryakova, Igor B. Rogozin, Saulius Serva, Alexei Yu. Kostygov, Vyacheslav Yurchenko

**Affiliations:** a Life Science Research Centre, Faculty of Science, University of Ostrava, Ostrava, Czech Republic; b Institute of Parasitology, Biology Centre, Czech Academy of Sciences, České Budějovice (Budweis), Czech Republic; c Faculty of Science, University of South Bohemia, České Budějovice (Budweis), Czech Republic; d Laboratory of Genetics, Institute of Botany, Nature Research Centre, Vilnius, Lithuania; e Department of Pathophysiology and Allergy Research, Center of Pathophysiology, Infectiology and Immunology, Medical University of Vienna, Vienna, Austria; f National Center for Biotechnology Information, National Library of Medicine, National Institutes of Health, Bethesda, Maryland, USA; g Department of Biochemistry and Molecular Biology, Institute of Biosciences, Vilnius University, Vilnius, Lithuania; University at Buffalo

**Keywords:** *Leishmania guyanensis*, *Leishmania major*, LRV1, LRV2, capsid

## Abstract

Leishmaniaviruses (LRVs) have been demonstrated to enhance progression of leishmaniasis, a vector-transmitted disease with a wide range of clinical manifestations that is caused by flagellates of the genus *Leishmania*. Here, we used two previously proposed strategies of the LRV ablation to shed light on the relationships of two *Leishmania* spp. with their respective viral species (*L. guyanensis*, LRV1 and L. major, LRV2) and demonstrated considerable difference between two studied systems. LRV1 could be easily eliminated by the expression of exogenous capsids regardless of their origin (the same or distantly related LRV1 strains, or even LRV2), while LRV2 was only partially depleted in the case of the native capsid overexpression. The striking differences were also observed in the effects of complete viral elimination with 2'C-methyladenosine (2-CMA) on the transcriptional profiles of these two *Leishmania* spp. While virtually no differentially expressed genes were detected after the LRV1 removal from *L. guyanensis*, the response of L. major after ablation of LRV2 involved 87 genes, the analysis of which suggested a considerable stress experienced even after several passages following the treatment. This effect on L. major was also reflected in a significant decrease of the proliferation rate, not documented in *L. guyanensis* and naturally virus-free strain of L. major. Our findings suggest that integration of L. major with LRV2 is deeper compared with that of *L. guyanensis* with LRV1. We presume this determines different effects of the viral presence on the *Leishmania* spp. infections.

**IMPORTANCE** *Leishmania* spp. represent human pathogens that cause leishmaniasis, a widespread parasitic disease with mild to fatal clinical manifestations. Some strains of leishmaniae bear leishmaniaviruses (LRVs), and this has been shown to aggravate disease course. We investigated the relationships of two distally related *Leishmania* spp. with their respective LRVs using different strategies of virus removal. Our results suggest the South American *L. guyanensis* easily loses its virus with no important consequences for the parasite in the laboratory culture. Conversely, the Old-World L. major is refractory to virus removal and experiences a prominent stress if this removal is nonetheless completed. The drastically different levels of integration between the studied *Leishmania* spp. and their viruses suggest distinct effects of the viral presence on infections in these species of parasites.

## INTRODUCTION

Leishmaniasis remains a public health concern affecting over 1.2 million people worldwide annually (1). It manifests in a repertoire of symptoms ranging from self-healing lesions in the case of cutaneous forms to fatal organ failures in visceral leishmaniasis (2). Even though the clinical picture of the disease usually depends on the infecting *Leishmania* species and the immune status of the host, our understanding of the molecular factors modulating the etiology of leishmaniases remains rather limited (3). One such a factor is the presence of double-stranded RNA (dsRNA) *Leishmania* RNA viruses (LRVs, genus *Leishmaniavirus*) of the family *Totiviridae*. Most totiviruses infect fungi (4), while some have been documented from animals (5–7) and protists (8–11). The LRVs suppress the anti-leishmanial immune response of the vertebrate host and, thus, provide a survival advantage to the parasites (12, 13). The two best studied species, LRV1 and LRV2, infect *Leishmania* of the New World (subgenus *Viannia*) and the Old World (subgenus *Leishmania*), respectively (14). Recently, two other *Leishmaniavirus* species, LRV3 and LRV4, have been described in *Blechomonas* spp., distant relatives of *Leishmania* parasitizing fleas (15). The dsRNA of LRV1 facilitates chronic inflammation and spread of *L. guyanensis* to secondary sites (16–18). It is generally assumed *Leishmania* and LRV coevolve (14, 19, 20), although occasional horizontal viral transfer events have also been reported (21). The genome of LRVs contains four open reading frames (ORFs), two of which (ORF2 and 3) encode the capsid and RNA-dependent RNA-polymerase (RDRP), respectively (22, 23).

As LRV presence is considered clinically important (24–26), different strategies of viral elimination were proposed in order to make *Leishmania* less virulent. One of the early approaches relied on hygromycin B treatment of *L. guyanensis*: parasites transfected with pX63-HYG plasmid and kept under antibiotic selection for several weeks lost the virus (27). The phenomenon was explained by specific inhibition of viral gene translation on the background of hygromycin B resistance of *Leishmania* strains. The resultant strain, *L. guyanensis* pX63-HYG, became a “gold standard” in all LRV1-related experiments for many years (16, 28). Another strategy was based on the chemical inhibition of viral replication by 2′C-methyladenosine triphosphate (2-CMA) (29, 30). Specific targeting of RDRP by this chemical led to the elimination of the virus without affecting *Leishmania* fitness. The last approach relied on an early observation that LRV2 capsid overexpression in L. major resulted in a significant and stable reduction of viral load (31). The self-assembled virus-like particles have inhibited *Leishmaniavirus* replication in a “dominant negative” manner; in other words, overexpression of native viral capsid proteins substantially interfered with essential processes in host cells. This is highly reminiscent of a description of this phenomenon in classical genetics (32). In line with the observed inhibition of replication, later studies have elegantly demonstrated LRV-facilitated leishmaniasis can be prevented by immunization with its viral capsid (33). A similar approach has been used to successfully eliminate L-A and M dsRNA viruses (family *Totiviridae*) of Saccharomyces cerevisiae (34–36).

In the current study, we systematically investigated the dominant negative effect of the capsid protein expression on the fate of LRVs in *L. guyanensis* and L. major, the specificity of the underlying mechanism, and the response of the flagellates to virus removal. While the elimination of LRV1 from *L. guyanensis* does not lead to any perceptible consequences for the parasite, LRV2 loss from L. major substantially changes the transcription profile and manifests in an attenuated cell division.

## RESULTS

### Establishment of the *L. guyanensis* lines expressing LRV1 capsid or its derivatives.

**FIG 1:**
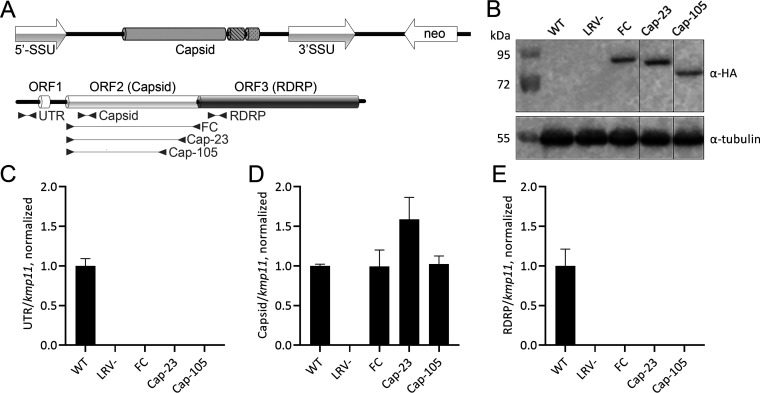
Expression of truncated capsids in *L. guyanensis* M4147. (A) Strategy for generation of the truncated capsids. Top: LRV1-4 integrated into the SSU-rRNA locus. Known capsid domains are represented by different shading and hatching. Bottom: genome organization of LRV1-4 with indicated open reading frames (ORF). Arrowheads indicate the RT-qPCR primer sets (“UTR,” “Capsid,” and “RDRP”) used in expression analyses and PCR primers used to generate wild-type (FC, full capsid), Cap-23, and Cap-105 constructs. (B) Western blotting confirmation of capsids’ expression. Sizes are in kDa. (C to E) RT-qPCR analysis of viral load and capsid expression in cultures overexpressing either full capsid or truncated capsid isoforms. Wild-type (WT) and *L. guyanensis* cured of virus (LRV–) were used as positive and negative controls, respectively. Data presented as normalized means and standard deviations of three independent biological replicates.

We employed the standard pLEXSY-based conventional approach to integrate genes encoding LRV1 capsid or its derivatives (Cap-23 and Cap-105) into the 18S rRNA locus of this species as in Zakharova et al. and Ishemgulova et al. (37, 38) (Fig. 1A). The successful integration and capsid expression were confirmed by genomic PCR (Fig. S1), Western blotting (Fig. 1B), and Reverse transcription-quantitative polymerase chain reaction (RT-qPCR) (Fig. 1C to E). As a negative control in RT-qPCR and Western blotting experiments, a cell line of *L. guyanensis* cured of LRV1 (labeled LRV1–) was used (29, 30). Notably, the expression of the rRNA-integrated capsids was comparable to that of the endogenous LRV1 as judged by the RT-qPCR analysis with primers annealing to both endogenous and exogenous capsid RNAs (“Capsid” in Fig. 1A lower panel and Fig. 1D).

10.1128/msphere.00335-22.1 FIG S1 PCR confirmation of the correct genomic integration for genes encoding LRV capsids and their derivatives. (A) Schematic representation of *Leishmania* spp. 18S rRNA locus depicting integration of the flanked Capsid sequence and PCR primers used to check for correct integration. (B) PCR confirmation of correct genomic integration for the full-length (FC) and truncated (Cap-23 and Cap-105) capsid sequences in *L. guyanensis* genome. Sizes on the left are in kb. (C) PCR confirmation of correct genomic integration of heterologous capsid sequences in *L. guyanensis* and L. major genomes. The lower panels in C and D shows DNA integrity control (PCR with primers detecting *kmp11*). Sizes on the left are in kb. Download FIG S1, PDF file, 0.1 MB. Copyright © 2022 Saura et al. 2022 Saura et al. https://creativecommons.org/licenses/by/4.0/ This content is distributed under the terms of the Creative Commons Attribution 4.0 International license.

### Overexpression of the full-length or truncated capsid eliminates LRV1 from *L. guyanensis*.

Next, we investigated the effect of exogenous capsid (or its derivatives) expression on the fate of endogenous LRV1. Similar to what has been reported for L. major and LRV2 (31), the expression of the full-length capsid of LRV1 has eliminated the endogenous virus from *L. guyanensis* (Fig. 1C and E). Note the primer sets used in these analyses (“UTR” and “RDRP”; Fig. 1A lower panel) detected only the endogenous LRV1. The same effect was documented for the Cap-23 and Cap-105 constructs (Fig. 1C and E). Of note, while the elimination of endogenous LRV2 from L. major in a previous study was only partial (31), the ablation of LRV1 from *L. guyanensis* was complete.

### Dominant-negative effect of the capsid overexpression on LRV1 and LRV2.

**FIG 2:**
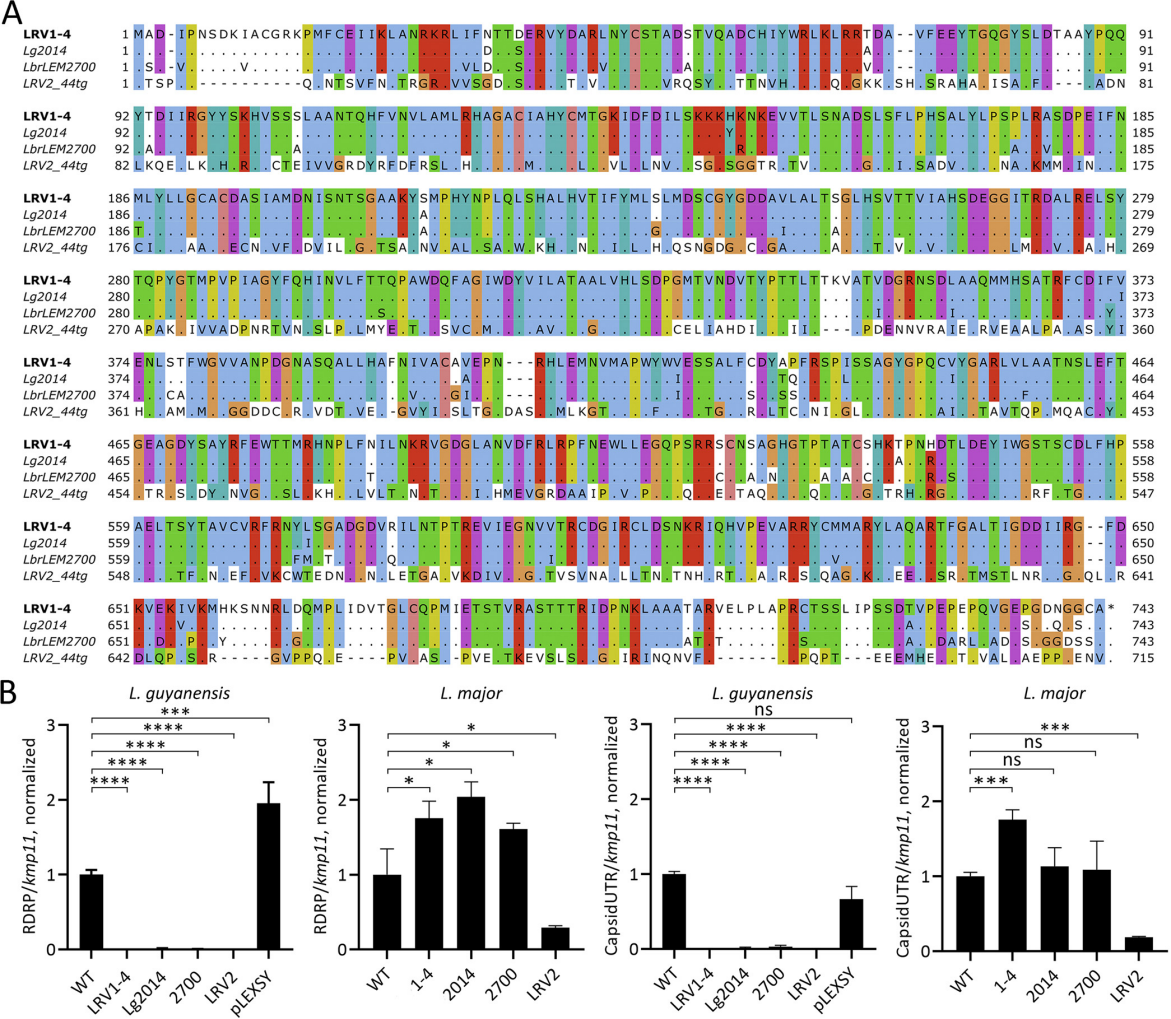
Dominant-negative effect of the capsid overexpression in different *Leishmania* spp. (A) Sequences of the LRV capsids, used in this work, aligned with MAFFT using G-INS-I method and visualized in Jalview using ClustalX color scheme. Amino acids identical to the top sequence are replaced with dots. (B) RT-qPCR analysis of viral load in *L. guyanensis* M4147 and L. major T44g cultures expressing different capsid proteins. WT, wild type; 1-4, LRV1-4 of *L. guyanensis* M4147; 2014, LRV1 of *L. guyanensis Lg*2014; 2700, LRV1 of *L. braziliensis Lbr*LEM2700; LRV2 of L. major T44g; pLEXSY, empty plasmid control. Data presented as normalized means and standard deviations of three independent biological replicates. *P-*values are denoted as follows: ns, not significant; *, *P ≤ *0.05; **, *P ≤ *0.01; ***, *P ≤ *0.001; ****, *P ≤ *0.0001.

We used two LRV-positive *Leishmania* spp. (*L. guyanensis* M4147 and L. major T44g) and overexpressed capsids of their own viruses (LRV1-4 and LRV2, respectively) as well as those of phylogenetically distant LRV1s from *L. guyanensis Lg*2014 and *L. braziliensis* LEM2700 (21). Successful integration and capsid expression were confirmed by RT-qPCR and Western blotting. While RNA levels of exogenous capsids were similar, the protein levels significantly varied up to the virtually undetectable in the case of LRV1 *Lbr*LEM2700 capsid expressed in L. major (Fig. S2). This suggests differential stability of the capsid proteins depending on their sequences (Fig. 2A) and *Leishmania* species/strain, caused, for example, the capsid proteins’ ability to trigger autophagy response in L. major (and not in *L. guyanensis*).

10.1128/msphere.00335-22.2 FIG S2 Expression of endogenous and exogenous LRV capsids in *L. guyanensis* M4147 and L. major T44g. (A) RT-qPCR analysis of RNA expression. Data presented as normalized means and standard deviations of three independent biological replicates. (B) Western blotting of capsid protein expression with anti-HA and anti-tubulin (loading control) antibodies. Sizes on the left are in kDa. Download FIG S2, PDF file, 0.2 MB. Copyright © 2022 Saura et al. 2022 Saura et al. https://creativecommons.org/licenses/by/4.0/ This content is distributed under the terms of the Creative Commons Attribution 4.0 International license.

Expression of all the exogenous (either LRV1 or LRV2) capsids eliminated the endogenous virus from *L. guyanensis* and none of them was able to exert the same effect in L. major. However, the LRV2 levels in L. major significantly decreased (*P* value ≤ 0.05) in the presence of additional LRV2 capsid (Fig. 2B).

### Whole-transcriptome analysis of virus-positive and -negative L. major and *L. guyanensis* strains.

Prompted by the data on stability of the LRV2-L. major T44g association, we decided to investigate whether the effect of LRV1/2 ablation is the same in *L. guyanensis* and in L. major. For that we used virus-negative isogenic lines of L. major T44g and *L. guyanensis* M4147 established by 2-CMA treatment. As a control, we also treated the natively virus-free strain L. major LV39 in the same way. After six passages with 2-CMA, followed by another six passages in drug-free medium, no LRV RNA could be detected by RT-qPCR in either of the three strains (Fig. S3).

10.1128/msphere.00335-22.3 FIG S3 Viral load in *L. guyanensis* M4147, L. major T44g, and L. major LV39 after six passages in 2-CMA (labeled 2-CMA) and after six passages in 2-CMA, followed by six passages in drug-free medium (labeled 2-CMA, released). RT-qPCR analysis of RDRP RNA expression. Data presented as normalized means and standard deviations of three independent biological replicates. Download FIG S3, PDF file, 0.1 MB. Copyright © 2022 Saura et al. 2022 Saura et al. https://creativecommons.org/licenses/by/4.0/ This content is distributed under the terms of the Creative Commons Attribution 4.0 International license.

**FIG 3:**
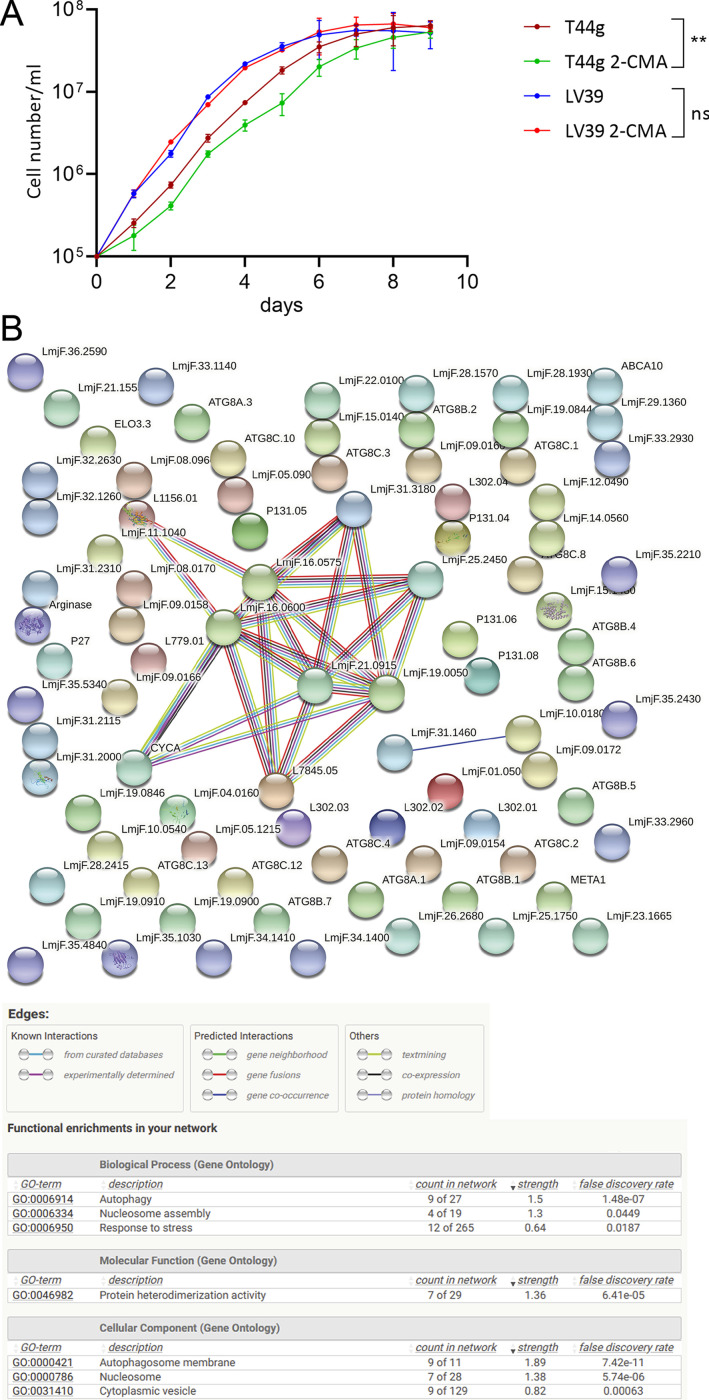
Comparison of virus-positive and virus-negative L. major T44g and *L. guyanensis* M4147. (A) Growth curves (see Materials and Methods for experimental details). **, *P* value ≤ 0.01. (B) STRING-based protein–protein network reconstruction. For the known interactions, turquoise line indicates those that came from the curated databases and crimson line indicates those that were experimentally determined; for predicted interactions, green line indicates gene neighborhood, red line indicates gene fusion, blue line gene cooccurrence, black line indicates coexpression, and light blue line indicates protein homology. Empty and filled nodes denote proteins with unknown and known or predicted three-dimensional (3D) structure, respectively. Data on functional enrichment (Biological process, Molecular function, and Cellular component) are tabulated at the bottom.

Previously, it has been demonstrated LRV1-4 ablation from *L. guyanensis* M4147 does not influence the culture growth (Fig. 2 in [29]). In contrast, our data demonstrated that elimination of LRV2 from L. major T44g results in a slower multiplication rate (*P* = 0.0078), although the density reached on day 9 was the same as in the wild-type strain. Notably, the L. major LV39 strain used as a control was dividing at a higher rate, which was not affected by 2-CMA treatment (Fig. 3A).

To get insight into the molecular mechanisms behind the differences in the response to viral removal, we compared whole transcriptome profiles of virus-positive and virus-negative L. major T44g and *L. guyanensis* M4147. We detected only two differentially expressed genes, which are downregulated in cells upon LRV1-4 ablation when comparing *L. guyanensis* M4147 cell lines with and without LRV1-4. These genes encode a putative subunit of the gamma-tubulin complex (*Lgu_360054900*) and a proteasome activator protein PA26 (*Lgu_350012100*). In stark contrast, 87 differentially expressed genes (67 and 20 up- and downregulated, respectively) were found in LRV-negative L. major T44g cells (Table S1). Notably, nine of the proteins, encoded by these genes (all upregulated), formed a well-defined cluster of interaction partners in the STRING analysis (Fig. 3B; number of nodes: 87; number of edges 24; PPI enrichment *P* value 7.59 × 10^−11^). We found all genes from this cluster are all upregulated with approximate fold change of ~2 ×. The gene ontology (GO) enrichment and KEGG analyses identified statistically significant overrepresentation of transcripts involved in cell response to various stimuli, autophagy, and nucleosome assembly among upregulated genes (Fig. S4), while expression of four zinc finger motif-contacting proteins, leucine-rich protein, cyclin, and arginase were found among 20 downregulated genes. The downregulation of cyclin is in a good agreement with the growth kinetics of 2-CMA treated L. major cells (Fig. 3A). Also, a reduced expression of the membrane-bound acid phosphatase 2 (Table S1) is noteworthy, as we have recently demonstrated ablation of a related enzyme (phosphatidate phosphatase 2-like protein) has profound effect in *Leishmania* biology (37).

10.1128/msphere.00335-22.4 FIG S4 Results of GO enrichment analysis for 67 genes, up-regulated in virus-negative L. major T44g. Enriched GO terms are grouped as follows: MF, molecular function; BP, biological process; CC, cellular component. The L. major gene names listed in columns; color encoding reflects annotation type: orange, gene model inferred from sequence alignment or gene orthology; blue, gene model was computationally predicted. Genes involved in KEGG pathway “autophagy” are marked black in the bottom panel. Download FIG S4, PDF file, 0.9 MB. Copyright © 2022 Saura et al. 2022 Saura et al. https://creativecommons.org/licenses/by/4.0/ This content is distributed under the terms of the Creative Commons Attribution 4.0 International license.

10.1128/msphere.00335-22.5 TABLE S1 Genes differentially expressed between virus-positive and virus-negative L. major T44g. Download Table S1, XLSX file, 0.01 MB. Copyright © 2022 Saura et al. 2022 Saura et al. https://creativecommons.org/licenses/by/4.0/ This content is distributed under the terms of the Creative Commons Attribution 4.0 International license.

Thus, the ablation of LRV2 from Leishmania major T44g has more dramatic outcomes than elimination of LRV1-4 from *L. guyanensis* M4147. This also correlates with the fact that LRV1-4 (but not LRV2) could be completely eliminated by overexpression of capsid of either LRV1 or LRV2 origin.

## DISCUSSION

The relationships between the LRVs and their flagellate hosts have been previously assessed regarding the importance of the viruses for the virulence of *Leishmania* spp. (16–18, 39, 40). Here, we attempted to get an insight into these associations from a different perspective: the extent of mutual adaptation within them. For this purpose, we used two different strategies of viral removal, which have been proposed before: dominant negative effect by capsid overexpression and 2-CMA treatment. The first approach is based on the disruption of the ideal 120:2 ratio between the capsid and fused (capsid-RDRP) protein, which results in a decreasing proportion of correctly assembled virions and subsequent viral loss (31, 41). We also tested two C-terminally truncated versions that preserved all functional capsid elements (21, 42), because it was demonstrated virus loss can be observed even with an abridged capsid protein in the case of the related L-A virus of yeasts (35). The results in the *L. guyanensis-*LRV1 system led to complete viral elimination, regardless of the experimental construct used, suggesting the C-terminal part of the capsid protein is not necessary for the assembly. Moreover, such an effect could be achieved even with nonnative capsids, which originated either from distantly related LRV1 strains, or, unexpectedly, another viral species—LRV2. These results imply the specificity of the capsid protein interactions is rather limited. In contrast, all the tested exogenous capsids were not able to remove LRV2 from L. major. Nevertheless, exogenous expression of the native capsid resulted in a significant decrease in LRV2 level in this species, although apparently to a lesser extent than was reported before for L. major strain MHOM/SU/73/5-ASKH (31). Importantly, it was previously demonstrated the decrease in the viral load was associated with LRV absence from the majority of leishmanial cells. The refractoriness of L. major to viral removal suggests a more intimate and stable relationship of this species with its virus than that of *L. guyanensis* with LRV1 or differences in autophagy-related response to exogenous capsids in these two species. This is further supported by the observation that while the growth of *L. guyanensis* after viral elimination remained unaffected, this resulted in a significant decrease of the proliferation rate in L. major. It appears virus removal is deleterious to L. major and, as judged by the transcriptome analysis, cells experience considerable stress even after several passages following the loss of the virus. This is rather surprising because until now the main advantage of bearing LRVs was regarded to be associated with the interaction of viruses with the immune system of vertebrate hosts, enhancing the progression of the infection (43). Indeed, it is unclear how the presence of a virus can be important under *in vitro* conditions. The most plausible explanation is the deep L. major-LRV2 integration. The usefulness of the virus for the fitness of L. major is questionable given a considerable proportion of virus-free strains in natural populations (21, 44). Regardless of the exact nature of this relationship, virus-bearing L. major seemingly tuned its cellular processes to coexist with the virus and removal of the latter apparently causes a “phantom pain,” i.e., disturbs an established balance.

Interestingly, LRV2 of L. major compared with LRV2 from *L. aethiopica* or LRV1s from *L. guyanensis* and *L. braziliensis* demonstrates a higher ratio of nonsynonymous to synonymous substitutions in both capsid and RDRP proteins. This pattern suggests a few sites in these proteins may be under positive selection (21). Therefore, the LRV2s may also intensively adapt to various strains.

The phenomenon of tight LRV2–L. major association discovered here poses new questions on the exact underlying molecular mechanisms, which deserve a further scrutiny. It is possible there are continuous or accidental interactions between virus proteins and the host genome transcription/translation processes that cause substantial changes in gene expression. This, in turn, should have an essential effect on L. major interactions with its sandfly vectors and mammalian hosts. Thus, our study highlights the profound difference between *L.* guyanensis-LRV1 and L. major–LRV2 associations, which presumably determines different impacts of the viral presence on the virulence of these leishmaniae.

## MATERIALS AND METHODS

### Strains, cultivation, viral elimination, and growth kinetics.

The wild-type strains *Leishmania guyanensis* MHOM/BR75/M4147 (in the text referred to as M4147, LRV1-positive [45]), L. major MRHO/UZ/2003/IsvT44g (in the text referred to as T44g, LRV2-positive [21]), and L. major MRHO/UZ/59/P (in the text referred to as LV39, virus-negative [16]) were cultivated in M199 (MilliporeSigma, Burlington, USA) supplemented with 2 μg/mL hemin (Jena Bioscience, Jena, Germany), 10% heat-inactivated fetal bovine serum (FBS, BioSera Europe, Nuaillé, France), 2 μg/mL biopterin, 100 units/mL of penicillin, and 100 μg/mL of streptomycin (all from Life Technologies/Thermo Fisher Scientific, Carlsbad, USA) at 23°C. Total genomic DNA was isolated from 5 × 10^7^ cells using GeneJET Genomic DNA purification kit (Thermo Fisher Scientific, Carlsbad, USA) and used for 18S rRNA gene amplification and species identity confirmation as described previously (46).

To cure viruses from *L. guyanensis* and L. major, cells of the virus-bearing strains were passaged six times in Schneider’s Drosophila medium supplemented with 10% FBS, penicillin, streptomycin as above, and 10 mM 2-CMA (29, 30). The virus-negative strain L. major LV39 was used as a specificity control in the 2-CMA treatment experiments. To evaluate the efficiency of elimination, the viral load was assayed by quantitative reverse transcription-PCR (RT-qPCR, see below) after six recovery passages in chemical-free medium. Afterwards, the parasites were cultured in complete M199, as defined above. Growth kinetics were analyzed for 9 days from a starting density of 1 × 10^5^ parasites per milliliter. Cell number was counted using a hemocytometer every 24 h as described previously (47) in three biological replicates for each strain/condition.

### Genetic manipulations and transfections.

Three LRV1 capsid-containing constructs for integration into *L. guyanensis* 18S rRNA locus were designed based on the position of known structural elements (42, 48): the full-length capsid (FC) ended at the frameshift, a Cap-23 variant terminated 23 amino acids upstream of it, and a Cap-105 version terminated immediately downstream of the annotated functional domains. See Table S2 for all primer sequences. These three capsid sequences were amplified from cDNA of *L. guyanensis* M4147 and cloned into pLEXSY-Neo2.1 (Jena Bioscience). The same was done for the full-length capsid sequence of LRV2 from L. major T44g. In addition, the LRV1 capsid sequences from *L. guyanensis Lg*2014 (NCBI accession number KY750611, labeled 2014) and *L. braziliensis Lbr*LEM2700 (NCBI accession number KX808483, labeled 2700) were synthesized at GeneCust (Boynes, France), and cloned directly into pLEXSY-Neo2.1. The choice of *Lg*2014 and *Lbr*LEM2700 was determined by their phylogenetic remoteness from LRV1-4 of *L. guyanensis* M4147 (labeled 1–4) (21).

10.1128/msphere.00335-22.6 TABLE S2 Primers used in this work. Download Table S2, XLSX file, 0.01 MB. Copyright © 2022 Saura et al. 2022 Saura et al. https://creativecommons.org/licenses/by/4.0/ This content is distributed under the terms of the Creative Commons Attribution 4.0 International license.

For transfection, 5 × 10^7^ *Leishmania* spp. cells were electroporated with 2 to 5 μg of SwaI-linearized plasmids using Nucleofector-2b (Lonza Bioscience, Basel, Switzerland) and program X-001. Transfected cells were incubated in complete M199 medium at 23°C: initially without antibiotic for 16 h and then with 50 to 100 μg/mL of Neomycin (VWR, Radnor, USA) for 3 weeks.

### Isolation of RNA, cDNA synthesis, and RT-qPCR.

Total RNA was isolated from 5 × 10^7^ cells in three biological replicates using the RNeasy minikit (Qiagen, Hilden, Germany) following the manufacturer’s recommendations. The cDNA was synthesized with random hexamer primers using the Transcriptor First Strand cDNA Synthesis Kit (Roche Life Science, Penzberg, Germany) following the manufacturer’s instructions. Capsid and RDRP expression were measured by RT-qPCR as described previously (49) using LightCycler 480 (Roche Life Science). All experiments were performed in biological (three randomly selected populations) and technical triplicates. Expression levels of genes of interest were normalized to the housekeeping gene kinetoplast membrane protein-11 (KMP11) (50) and shown relative to the wild type.

### Western blotting.

To validate capsid expression in different *Leishmania* spp. populations, lysates from approximately 1 × 10^7^ cells were probed with anti-HA and anti-tubulin antibodies (both from MilliporeSigma) at 1:1,000 and 1:5,000 dilutions, respectively, as in Kraeva et al. (51).

### Differential expression analysis of LVR-ablated and wild-type *Leishmania* spp.

Transcriptomes of L. major T44g wild-type and LRV2-ablated cells after six passages in 2-CMA-free media were sequenced in four independent biological replicates each. Transcriptomes of *L. guyanensis* M4147 wild-type and LRV1-ablated cells after six passages in 2-CMA-free media were sequenced in two series yielding seven independent biological replicates for each line in total. All samples were sequenced in paired-end mode on Illumina NovaSeq with read length of 150 bp. Reads were trimmed with Trimmomatic v. 0.39 (52), using “SLIDINGWINDOW: 10:25” and “TRAILING: 25” trimming functions and mapped on reference genome sequence with Bowtie2 (53) using “–sensitive-local –no-unal.” The reference sequence and annotation for L. major (strain Friedlin) were taken from the TriTrypDB release 54 (54). The reference sequence and annotation for *L. guyanensis* (strain 204) were downloaded from the NCBI (accession number GCA_003664525). Read counting was performed with BEDTools v. 2.30 (55). Analysis of differential expression was performed in R v. 3.5.1 with EdgeR package v. 3.26 (56), genes with overall low counts were filtered out with “filterByExpr(min.count =10, min.total.count =30)” function. Differentially expressed gene lists were generated using FDR-corrected *P*-value cut-off 0.01 and fold change cut-off 2. GO enrichment analysis was done with the g:Profiler2 v. 0.2.1 package (57).

### Network reconstruction.

The obtained differentially expressed genes were incorporated into the STRING v. 11.5 (58) and network reconstruction was performed on the basis of the corresponding proteins. Specific and meaningful protein–protein associations were indicated by edges. The interactions from the curated databases and those that were experimentally determined were included. The protein–protein interactions (PPI) enrichment *P* value was used to verify whether observed number of edges is significant and the nodes are not random.

### Statistical analyses.

The statistical analyses were performed using GraphPad Prism v. 9 (GraphPad Software, San Diego, USA). The two-tailed Student’s *t* test was applied for the analysis of RT-qPCR data. The growth curves were analyzed using two-tailed Wilcoxon matched-pairs signed rank test.
