# Supplementary figures and images for: Elimination of LRVs Elicits Different Responses in Leishmania spp
Source: mSphere. 2022 Aug 9;7(4):e00335-22. doi: 10.1128/msphere.00335-22 (PMC9429963; doi:10.1128/msphere.00335-22)

**A**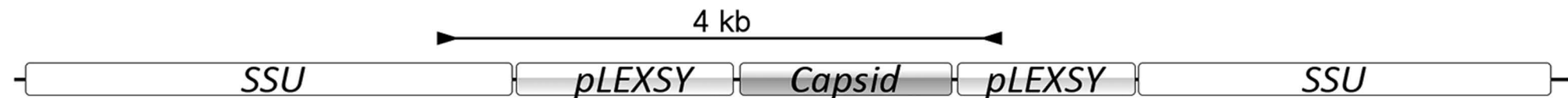**B**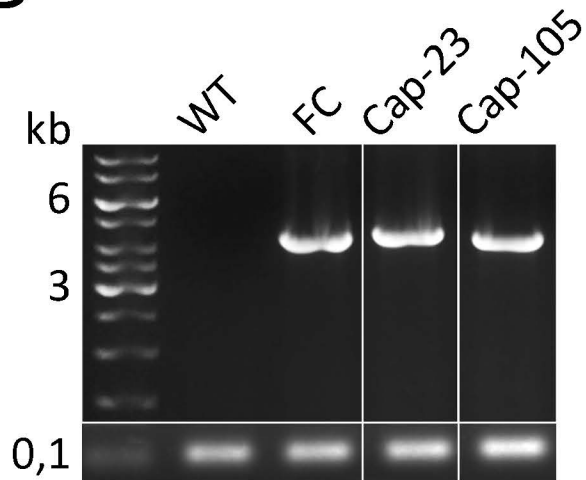**C**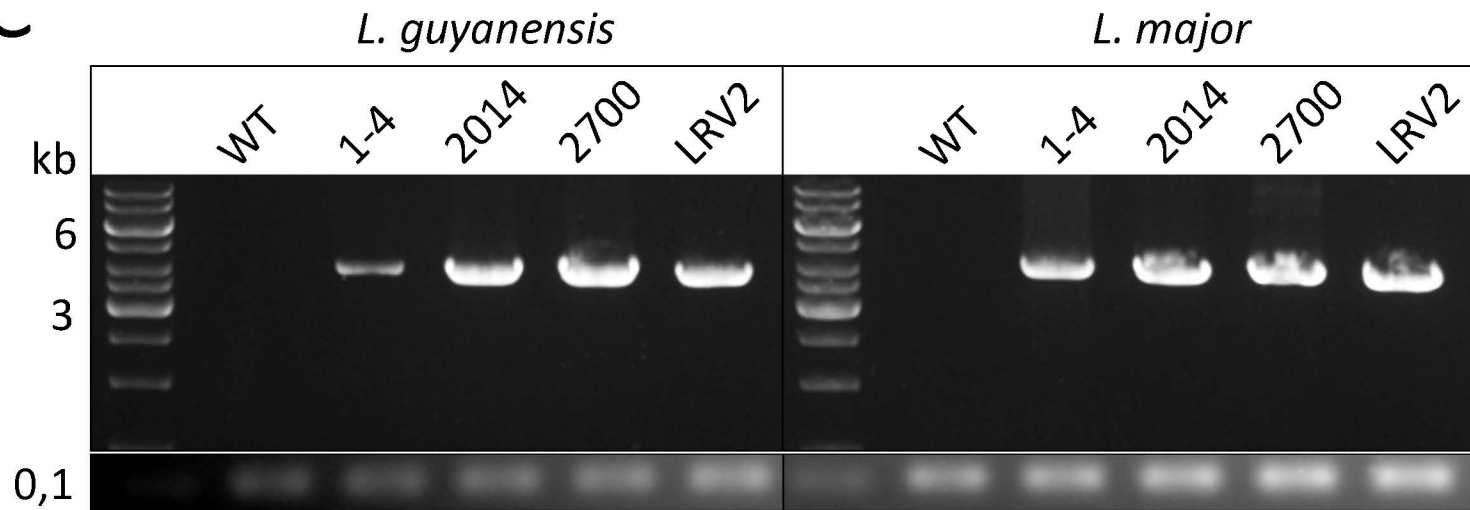

Supplement: FIG S1 [file msphere.00335-22-s0001.pdf]

**A**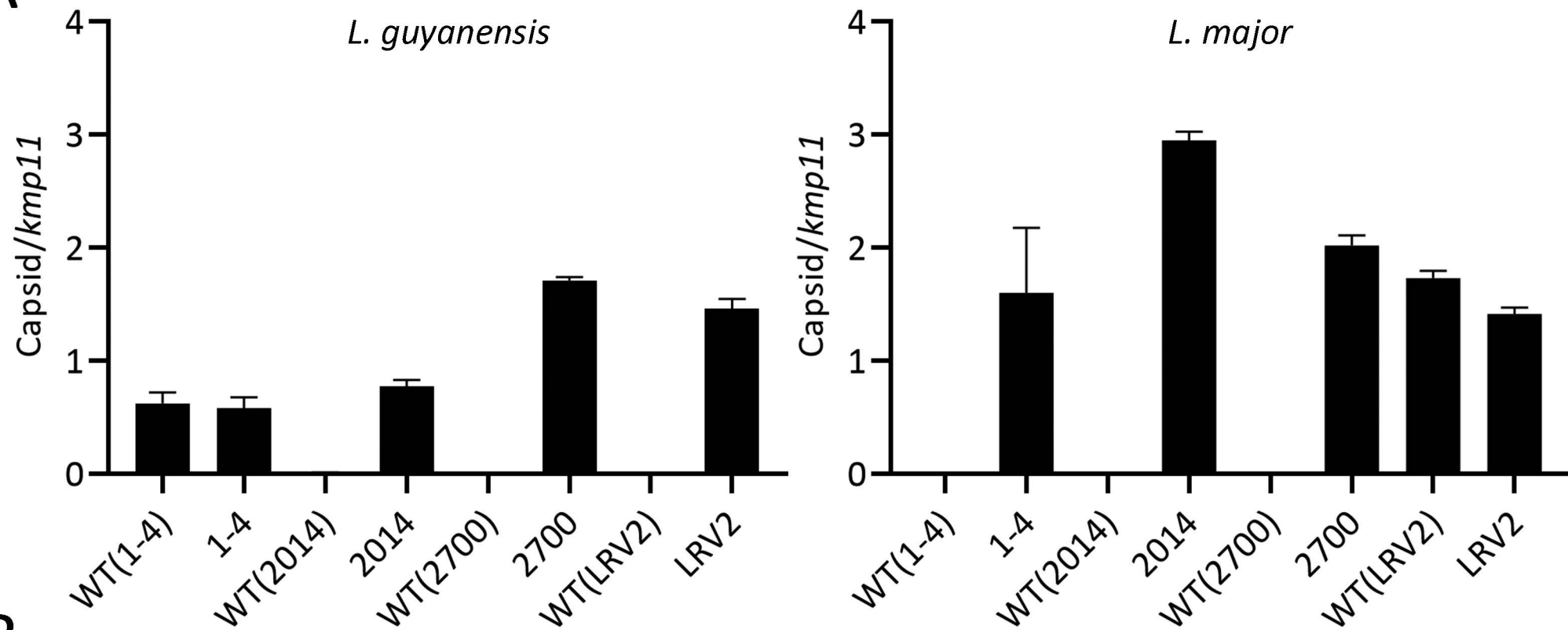**B**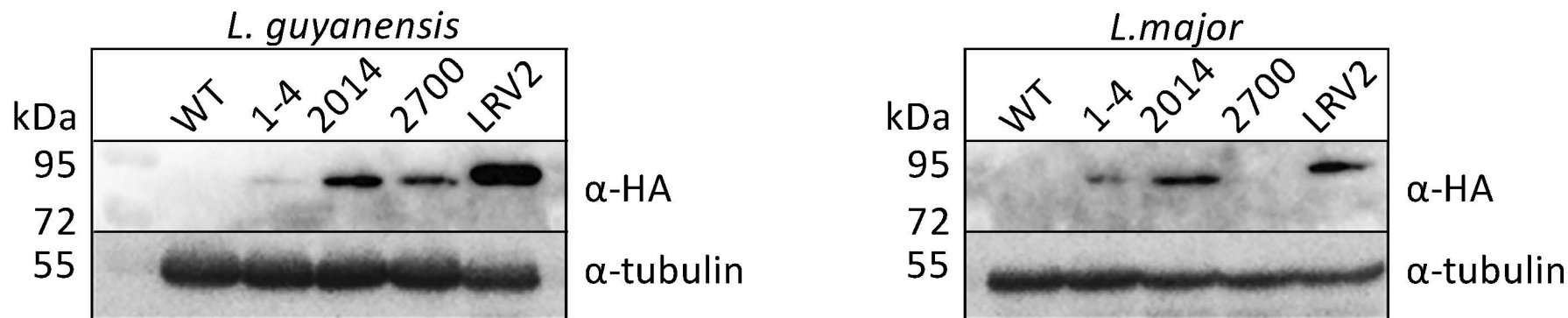

Supplement: FIG S2 [file msphere.00335-22-s0002.pdf]

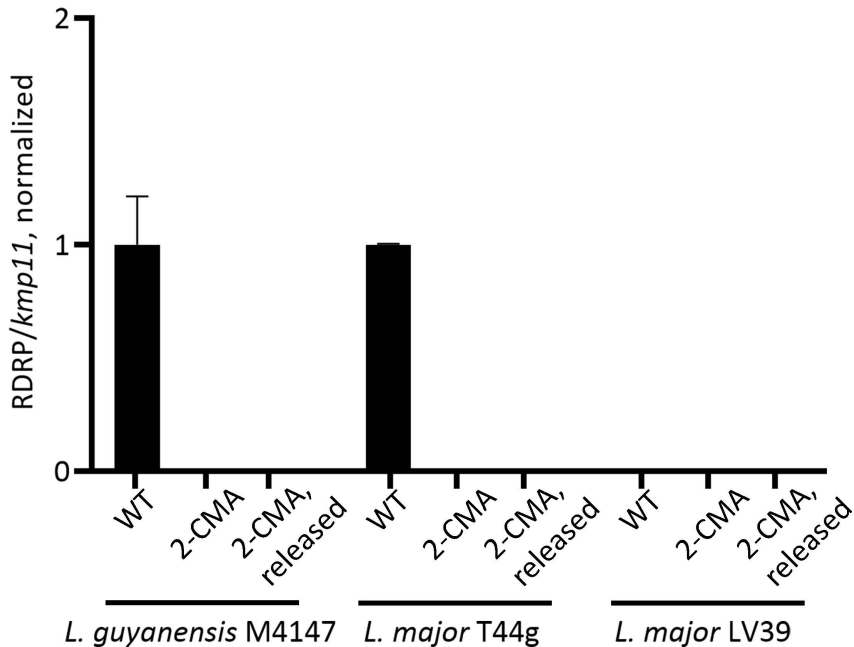

Supplement: FIG S3 [file msphere.00335-22-s0003.pdf]
