# Supplementary material for: Elimination of LRVs Elicits Different Responses in Leishmania spp
Source: mSphere. 2022 Aug 9;7(4):e00335-22. doi: 10.1128/msphere.00335-22 (PMC9429963; doi:10.1128/msphere.00335-22)
Supplement: FIG S4 [file msphere.00335-22-s0004.pdf]

GO:MF

Term name	Term ID	p_{adj}
protein heterodimerization activity	GO:0046982	3.432×10^{-8}
protein dimerization activity	GO:0046983	3.748×10^{-7}
metalloendopeptidase activity	GO:0004222	3.230×10^{-6}
metallopeptidase activity	GO:0008237	8.020×10^{-5}
endopeptidase activity	GO:0004175	4.113×10^{-3}
DNA binding	GO:0003677	9.930×10^{-3}
histone binding	GO:0042393	1.135×10^{-2}
peptidase activity	GO:0008233	3.109×10^{-2}

1 to 8 of 8 Page 1 of 1

GO:BP

Term name	Term ID	p_{adj}
cellular response to nitrogen starvation	GO:0006995	3.083×10^{-45}
cellular response to nitrogen levels	GO:0043562	3.083×10^{-45}
cellular response to extracellular stimulus	GO:0031668	4.771×10^{-41}
response to extracellular stimulus	GO:0009991	4.771×10^{-41}
response to nutrient levels	GO:0031667	4.771×10^{-41}
cellular response to nutrient levels	GO:0031669	4.771×10^{-41}
response to starvation	GO:0042594	4.771×10^{-41}
cellular response to starvation	GO:0009267	4.771×10^{-41}
cellular response to external stimulus	GO:0071496	5.403×10^{-40}
response to external stimulus	GO:0009605	9.011×10^{-38}
autophagy	GO:0006914	2.048×10^{-34}
process utilizing autophagic mechanism	GO:0061919	2.048×10^{-34}
cell communication	GO:0007154	3.335×10^{-19}
cellular response to stress	GO:0033554	7.192×10^{-17}
response to stress	GO:0006950	5.175×10^{-15}
cellular catabolic process	GO:0044248	1.201×10^{-12}
cellular response to stimulus	GO:0051716	2.439×10^{-12}
catabolic process	GO:0009056	3.096×10^{-11}
response to stimulus	GO:0050896	1.012×10^{-10}
biological adhesion	GO:0022610	5.202×10^{-7}
cell adhesion	GO:0007155	5.202×10^{-7}
chromatin assembly	GO:0031497	3.477×10^{-3}
nucleosome assembly	GO:0006334	3.477×10^{-3}
chromatin assembly or disassembly	GO:0006333	6.409×10^{-3}
nucleosome organization	GO:0034728	7.725×10^{-3}
DNA packaging	GO:0006323	7.725×10^{-3}
chromatin organization	GO:0006325	3.359×10^{-2}
protein-DNA complex assembly	GO:0065004	4.587×10^{-2}

1 to 28 of 28 Page 1 of 1

GO:CC

Term name	Term ID	p_{adj}
nucleosome	GO:0000786	1.552×10^{-4}
DNA packaging complex	GO:0044815	2.328×10^{-4}
chromatin	GO:0000785	6.396×10^{-4}
protein-DNA complex	GO:0032993	8.507×10^{-4}

1 to 4 of 4 Page 1 of 1

KEGG

Term name	Term ID	p_{adj}
Autophagy - other	KEGG:04136	3.620×10^{-19}

1 to 1 of 1 Page 1 of 1
